# How Do Brazilian Fires Affect Air Pollution and Public Health?

**DOI:** 10.1029/2020GH000331

**Published:** 2020-12-01

**Authors:** M. E. Marlier, E. X. Bonilla, L. J. Mickley

**Affiliations:** ^1^ Department of Environmental Health Sciences, Fielding School of Public Health University of California Los Angeles Los Angeles CA USA; ^2^ John A. Paulson School of Engineering and Applied Sciences Harvard University Cambridge MA USA

## Abstract

Fires burning across the Amazon in the summer of 2019 attracted global attention for the widespread destruction of natural ecosystems and regional smoke production. Using a combination of satellite fire observations and atmospheric modeling, Nawaz and Henze (2020, https://doi.org.10.1029/2020GH000268) provide new evidence for the widespread regional public health consequences attributed to these fires. They find that approximately 10% of premature deaths in Brazil due to fine particulate matter (PM_2.5_) are attributable to smoke pollution and highlight how fire locations play a critical role in determining downwind health impacts.

## Key Points

- Nawaz and Henze (2020) estimate the influence of the 2019 fire season in Brazil on air pollution and public health
- Future work is needed to link health outcomes to fire sources and explore interactions with overlapping health crises such as COVID‐19

## 1 Deforestation and Fire Activity in the Brazilian Amazon

Satellites have monitored historical deforestation and associated fire activity in Brazil for decades, but we are just beginning to understand the consequences for air pollution and public health. More than one million square kilometers (~13%) of natural forest cover was lost across Brazil from 1985 to 2019 (MapBiomas Project, 2020). In 2004, the Brazilian government implemented new regulations to reduce illegal deforestation in order to sustainably develop the region. Between 2005 to 2013, deforestation rates in the Brazilian Amazon declined by 70% as a result of these regulations, but has since steadily increased (Nepstad et al., 2014). The National Institute of Space Research in Brazil estimated that roughly 10,000 km^2^ of the Legal Amazon was cleared between July 2018 and August 2019, a 34% increase from the year before (INPE, 2020).

Fire activity in the Amazon region takes place mainly during the dry season, between August to November. During this period, fires are deliberately set to clear the dead vegetation leftover from prior deforestation, when land was cleared for human activities—e.g., mining, logging, and agricultural land use. Using visibility observations as a proxy for fire activity, van Marle et al. (2017) attributed the increase of fires in the Amazon starting in the 1990s to deforestation and forest degradation. Recent studies also found that the duration of the dry season is lengthening; frequent drought years (2005, 2010, and 2015) may also increase the incidence of fires in the Amazon (Aragão et al., 2014, 2018; Brando et al., 2019; Marengo et al., 2017).

Fires emit fine particulate matter (PM_2.5_) and trace gases that can lead to regional air pollution. For example, during the burning season, observed PM_2.5_ concentrations increased from 2 to 30–50 μg/m^3^ in the southwestern Amazon region (Reddington et al., 2019). Emerging evidence shows that smoke pollution from fires has a deleterious effect on human health (Cascio, 2018; Liu et al., 2015; Reid et al., 2016). In South America, previous modeling studies have estimated that regional fires are responsible for thousands of premature deaths per year (Butt et al., 2020; Johnston et al., 2012; Reddington et al., 2015).

## 2 Estimating the Influence of Fires on Air Pollution in Brazil

In their study, Nawaz and Henze (2020) quantify the premature deaths associated with smoke pollution from fires between July and September 2019 and compare to recent years. News reports indicated that the impacts from these fires were substantial. For example, smoke darkened skies in São Paulo thousands of kilometers away. Satellite imagery show that many of the 2019 fires were located near highways with newly deforested land (Voiland, 2019).

Fire activity is mapped with two different satellite‐based emissions inventories, the Quick Fire Emissions Dataset (QFEDv2.4) (Darmenov & da Silva, 2015) and the Fire Inventory from NCAR (FINNv1.5) (Wiedinmyer et al., 2011). The GEOS‐Chem adjoint is used as a computationally efficient method for determining the sensitivity of annual average population‐weighted PM_2.5_ concentrations to carbonaceous aerosol emissions (organic and black carbon; OC and BC) from fires across Brazil (Henze et al., 2007). This method links fire emissions with atmospheric transport and deposition processes to produce sensitivities of pollution to the location and magnitude of fires. Total exposure is estimated with a combination of the forward GEOS‐Chem model and satellite observations for 2010 as in Shaddick et al. (2018), with the 2016–2019 contributions from fires provided by the adjoint. Total and smoke PM_2.5_ is then used to estimate health outcomes using the Global Burden of Disease framework.

Fire emissions increased by 115% (1.37 Tg) from 2019 compared to 2018. Further, Nawaz and Henze (2020) estimate that smoke PM_2.5_ exposure from fires across Brazil contributed to 4,966 premature deaths in the fire season of 2019. This represented a 74% increase in mortality over 2018 and is slightly lower that the highest total in their study period (5,273 deaths in 2017). Across Brazil, July to September fires contributed to approximately 10% of total PM_2.5_‐related mortality. However, since the dry season in the Amazon typically ends in November, smoke emissions from the 2019 fires likely played an even greater role than was estimated in this study.

Nawaz and Henze (2020) emphasize the importance of considering interactions between the location of fires, atmospheric transport patterns, and where people live. One key advantage of their modeling framework is the ability to quantify the disproportionate impact of specific fires on smoke PM_2.5_. For example, fires upwind of population centers will have a larger impact on country‐level public health outcomes even if the overall magnitude of emissions is lower. The regions that contributed the most to the mortality burden are in southeastern Brazil, which encompasses several large cities. Emissions from central Brazil, however, have less of an influence on pollution in urban centers and on national health. Taken further, emissions from the areas with larger fires (>50 Gg OC + BC) contributed to ~14% of the mortality burden. Areas with high mortality burdens (>50 premature deaths), on the other hand, contributed more than 60% of the total mortality burden, even though such areas contributed just ~28% of total fire emissions. Since the adjoint model calculates population‐weighted health outcomes, the analysis emphasizes fires that contribute to smoke pollution in urban areas. Butt et al. (2020), in contrast, show that the less densely populated western states of Brazil are more heavily impacted by smoke pollution, although population levels in these locations are much lower.

Direct comparisons with other studies are challenging. A recent study by Butt et al. (2020) estimated that vegetation fires in the Amazon basin from August to October 2012 were linked to ~9,800 premature deaths in Brazil. Earlier, Reddington et al. (2015) estimated that ~7,000–9,800 and ~4,200–5,200 premature deaths annually across South America and Brazil from 2002 to 2011 were due to fires in Brazil. In addition to differences in the time period selection, spatial resolution of the atmospheric models, spatial domain, and fire emissions inventories, the studies use different concentration‐response functions to relate smoke PM_2.5_ to mortality. Butt et al. (2020) applied a concentration response function from the Global Exposure Mortality Model, which has higher risk estimates than the 2016 Global Burden of Disease estimates applied by Nawaz and Henze (2020). Reddington et al. (2015) focused on cardiopulmonary disease and lung cancer using a log‐linear concentration response function. In addition to mortality outcomes, the 2019 fires were linked to increased hospitalizations for respiratory illnesses, primarily for young children and elderly adults (Human Rights Watch, 2020).

Nawaz and Henze (2020) describe several key sources of uncertainty, including application of temporally averaged emissions sensitivity maps to calculate the total health impact of smoke PM_2.5_ since adjoint sensitivities are not available for 2019, the selection of fire emissions inventories, use of 2010 observations to constrain the surface PM_2.5_, and the relatively coarse horizontal resolution of their model. Other uncertainties stem from the absence of a detailed validation of the surface PM_2.5_ concentrations calculated by the model. In addition, the version of the GEOS‐Chem adjoint model used to estimate smoke PM_2.5_ does not take secondary organic aerosol (SOA) into account, even though observations suggest that ~ 40% of total carbon particulate mass in the Amazon can be traced to SOA (e.g., Gilardoni et al., 2011). Nawaz and Henze (2020) calculate premature mortality with and without the contribution of fire emissions and take the difference to estimate the smoke contribution. Given that the health response to PM_2.5_ flattens at high particle concentrations (Cohen et al., 2017), this implied underestimate in background PM_2.5_ could lead, in turn, to an overestimate of premature mortality due to smoke PM_2.5_ close to source regions. On the other hand, a recent meta‐analysis found larger‐than‐expected impacts on premature mortality at low levels of PM_2.5_ (Vodonos et al., 2018), implying that the health impacts of smoke PM_2.5_ in regions far from fire locations may be underestimated. Nawaz and Henze (2020) estimate mortality based on annual exposure, despite the more episodic nature of fires that occur over the course of several. Recent studies in the United States and Canada suggest that incorporating short‐term health outcomes would increase the total health burden due to fires (Fann et al., 2018; Liu et al., 2017; Matz et al., 2020). Finally, Nawaz and Henze (2020) also assume that the health impact of smoke is comparable to that of other particulates (Cascio, 2018).

## 3 Suggested Future Work

We suggest several directions for future work. Nawaz and Henze (2020) do not consider the influence of individual fire types on the downwind pollution burden or transboundary pollution. An extension of work by Reddington et al. (2015), which isolated the impact of deforestation and degradation‐related fires on smoke pollution, would be useful for determining effective policy options to improve air quality. In addition, the mortality burden from smoke PM_2.5_ exposure calculated by Nawaz and Henze (2020) mainly accounts for densely populated region in western and southern Brazil but notably does not distinguish between the health effects on urban populations and those on the indigenous peoples or rural populations living in close proximity to these fires. Continued increases in smoke would affect these already vulnerable communities who lack access to proper healthcare and exhibit high rates of respiratory illnesses (Cardoso et al., 2010; Geraque, 2020; Gracey & King, 2009; Portela et al., 2005). Future work could also incorporate health outcome data previously developed for the exposure of Brazilian populations to biomass burning smoke pollution (Ignotti et al., 2010).

In addition to air pollution, fires in the Amazon can threaten the socioeconomic security of the broader region. One example is that smoke from the fires in the Amazon can travel thousands of kilometers, with satellite observations suggesting that at least some smoke is transported across the Andes (Bourgeois et al., 2015). Schmitt et al. (2015) hypothesized that some of the black carbon detected in Andean snow pack and glaciers may have originated from fires in the Amazon. By reducing the surface albedo of Andean glaciers, the smoke could thus contribute to their observed rapid melting and impact communities in Peru and Bolivia that rely on the spring runoff.

## 4 Relevance to the 2020 Fire Season and Beyond

**Figure 1:**
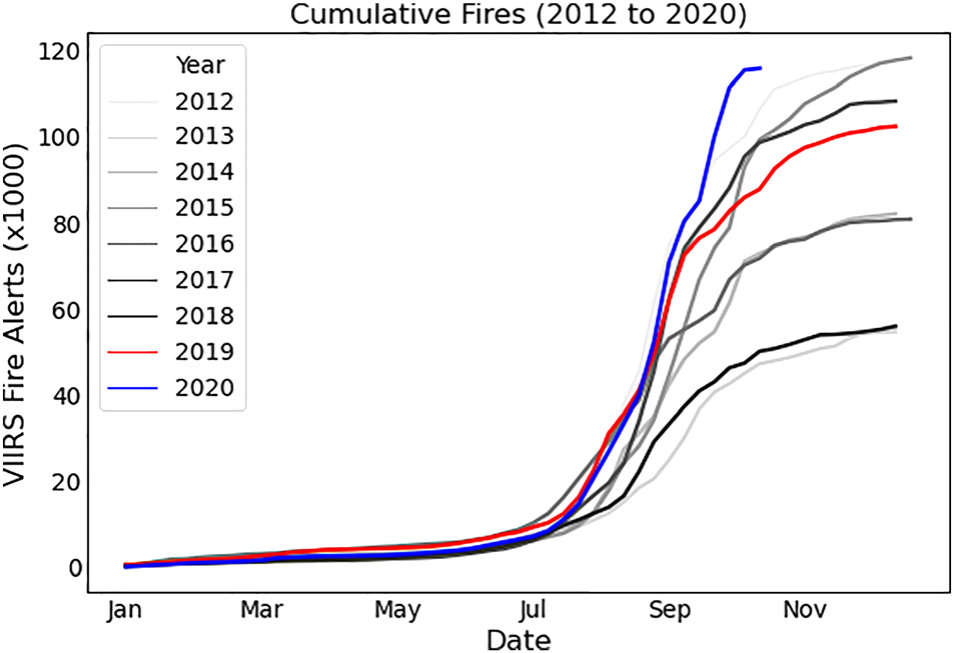
Cumulative weekly fire alerts in Brazil from the Visible Infrared Radiometer Suite (VIIRS) sensor for each year from 2012 to 2020 with 2019 (red), 2020 (blue; includes observations through mid‐October), and all other years in gray. Only high confidence fires are shown (Global Forest Watch, 2020).

For 2020, the picture of fire activity is still emerging. Across Brazil, satellite fire detections from the Visible Infrared Radiometer Suite (VIIRS) sensor show elevated fire activity through October 2020 compared to the same period in 2019 (Figure 1). Recent news reports suggest that reduced governmental oversight during the COVID‐19 pandemic may have further increased deforestation and biomass burning in Amazonia in 2020 (BBC, 2020; Roberton & Bodo, 2020). In addition, although the region experienced an uptick in deforestation in 2019, much of the vegetation felled that year has not yet burned (Goodman & Giles, 2020), leading to an overabundance of fuel that could readily ignite and potentially enhance the smoke burden in 2020 and subsequent years (Alencar et al., 2020).

As Nawaz and Henze (2020) point out, the increase in smoke exposure in 2019 in Brazil is concerning for public health. Looking towards the current season, recent ecological studies in the United States have suggested a link between exposure to high levels of total PM_2.5_ and increased COVID‐19 deaths (Rodriguez‐Diaz et al., 2020; Wu et al., 2020) while also considering the influences of population density, socioeconomic factors, and stay‐at‐home orders. While no study to our knowledge has yet examined the relationship between smoke PM_2.5_ exposure and COVID‐19 infection rates, Landguth et al. (2020) recently detected a significant relationship between exposure to wildfire smoke during the summer/early fall and rates of influenza infection during the following winter in Montana. A potential link between smoke PM_2.5_ and COVID‐19 has special relevance to public health in Brazil, where the rates of COVID‐19 infections are among the highest in the world, with ~4.5 million total confirmed cases and ~136,000 deaths as of mid‐September, 2020 (Johns Hopkins Coronavirus Resource Center, 2020), and vulnerable populations could be particularly susceptible (de Oliveira et al., 2020). Such a link could be important in other fire‐prone regions around the world facing extreme fire seasons during the COVID‐19 pandemic, including the western United States.

## 5 Conclusions

Nawaz and Henze (2020) estimate that nearly 5,000 premature deaths in Brazil during the 2019 fire season were attributable to fire emissions, a 74% increase over 2018. Using an adjoint modeling framework, they are able to determine which locations contribute the most to population‐weighted PM_2.5_ concentrations. Future work is needed to link these health outcomes to fire sources such as deforestation or degradation and explore interactions with overlapping health crises such as COVID‐19.

## Conflict of Interest

The authors declare no conflicts of interest relevant to this study.
